# The Impact of the COVID-19 Confinement on the Habits of PA Practice According to Gender (Male/Female): Spanish Case

**DOI:** 10.3390/ijerph17196961

**Published:** 2020-09-23

**Authors:** Marta García-Tascón, César Sahelices-Pinto, Cristina Mendaña-Cuervo, Ana María Magaz-González

**Affiliations:** 1 Department of Sports and IT, Faculty of Sport Sciences, University of Pablo de Olavide, 41013 Seville, Spain; 2 Department of Economic and Business Administration, Faculty of Economics and Business Administration, University of León, 24071 León, Spain; cesar.sahelices@unileon.es (C.S.-P.); cristina.mendana@unileon.es (C.M.-C.); 3 Department of Didactic of Musical, Plastic and Corporal Expression, Faculty of Education, Soria, University of Valladolid, 42004 Soria, Spain; anamaria.magaz@uva.es

**Keywords:** COVID-19, gender, physical activity, health

## Abstract

The declaration of the COVID-19 pandemic has resulted in drastic changes to life worldwide. In Spain, the state of alarm caused the confinement of 47 million inhabitants, affecting every aspect of life. This study analyzes the impact of such confinement on the health of men and women, as well as the effect on the practice of physical activity (PA) of both genders. An ad hoc questionnaire was administered. A total of 1046 people (48.57% men and 51.43% women) with an average age of 40 years (SD ± 13.35) participated in this study. For both genders, there was a significant decrease in quantity and intensity (*p* = 0.000). There was also an alteration in the type of PA practiced, shifting from cardiorespiratory exercise and muscular fitness to flexibility and neuromotor exercise (especially in women). The most popular way of practicing PA during the confinement was “autonomously” (statistically higher in men (M = 3.58) compared to women (M = 3.18)) and the most frequent format was “virtual” (statistically higher in women (M = 2.81) compared to men (M = 1.94)). Confinement modifies the habits of PA practice, especially in men. Both genders put their health and quality of life at risk by not following the PA guidelines of the health authorities World Health Organization (WHO) and American College of Sports Medicine ACSM). These conclusions highlight the importance of considering gender when designing programs and PA formats for the promotion of physical activity to reduce the existing gender divide.

## 1. Introduction

The World Health Organization (WHO) announced, at the end of January 2020, that the outbreak of a new coronavirus, known as SARS-CoV-2, posed a new, “alarming”, international public health emergency. Then, on 11 March, this organization declared COVID-19 as a global pandemic, causing the greatest global confinement in history, at different levels depending on the different governments [1].

Spain declared a state of alarm on 14 March through Royal Decree 463/2020 [2] and, consequently, the confinement of the entire Spanish population (47 million inhabitants). The state of alarm was prolonged five times until it was terminated on 21 June 2020 through RD 555/2020, on 5 June [3]. During this period, personal limitations of different degrees were established, limiting the freedom of movement to the home environment. The first month and a half was the most restrictive, since people could only leave their homes to cover their basic needs (art. 7 of RD 463/2020).

Order SND/380/2020, of 30 April [4], reduced the movement restrictions, made the confinement conditions more flexible and allowed people from 14 years of age to practice non-professional physical activity (PA) outdoors from 2 May 2020, with time limitations, individually and without contact.

This unprecedented and extraordinary scenario had a great impact on everyday life (closing of cities, schools, businesses, non-essential infrastructures, gyms, parks, restaurants, etc.). The structure of production and sales was altered abruptly, causing the contraction of almost all sectors; the temporary employment regulation files (TERFs) increased, reaching nearly 4 million in April 2020 [5], and the gross domestic product (GDP) decreased by 18.5% in the second trimester of 2020 in Spain and up to 11.90% in the European Union [6]. 

The serious health situation, the restriction of freedom of movement, the job and production transformation, the limits to social and personal contact and the pattern modification regarding food, sleep, socialization and PA practice had a direct impact on physical and emotional health [7], especially in women [8].

The sector of physical sports activity (PSA) was not outside of this context and it was altered in all its manifestations: postponement of the Olympic Games [9,10,11], suspension of national and international tournaments [12], closing of facilities, sports centers and outdoor spaces [13,14,15,16,17], with uncertain and concerning consequences [9,17,18,19,20] for the field of sports in general. The fitness sector estimates at least 44% less invoicing for the next twelve months, assuming losses of 1.108 billion euros [21]. TERFs have affected 98% of gyms and sports centers and at least 75–100% of their staff [17,19].

Some sports activities were maintained during the confinement thanks to information and communication technology (ICT), the digital immersion of many organizations in a very short time [16,22,23,24] and virtual initiatives of institutions [25,26]. However, the traditional behavior of practicers does not include the consumption of virtual PSA from their homes [27,28,29], since this behavior is rather motivated by the social relationship that accompanies this practice, which is one of the reasons that technological tools had not been in high demand before this time [30].

In general, the habits of sports consumption were modified during the confinement and PSA practice dropped sharply [14,15]. For instance, in Spain, during the weeks of confinement, there were 38% less steps per week with respect to the period before the confinement [31], the intensity and quantity of practice decreased [32], the type of PA performed was modified [29,32] and the practice scenarios changed [27]. 

Therefore, the confinement and the circumstances described may have modified the healthy standards of intensity, quantity and type of PA practice recommended by the health authorities [33,34,35,36], thus deteriorating the general health state and quality of life of Spanish men and women, with the probable increase in the cost derived from physical inactivity estimated at 1.8 billion euros for the year 2020 [19]. Indeed, performing no PA at all and having a sedentary lifestyle is associated with a high risk of mortality by cardiovascular disease, obesity, cancer, hypertension, type II diabetes, disability associated with non-communicable diseases (NCDs), depression and anxiety [33,37,38,39,40], among others. 

In fact, the scientific evidence supports the benefits of PA to decrease such diseases, to improve physical condition and quality of life, as a tool for cardio-oncological recovery [41], to strengthen and prepare the immune system in order to give a better response against communicable viral diseases such as COVID-19 [42] and to increase resilience and decrease depression [43,44]. Therefore, the health authorities recommend fast-pace walks, moderate-intensity aerobic activity, spending less time sitting [45,46] and vigorous exercise to strengthen the muscles [37,47,48,49].

The WHO [36] and the American College of Sports Medicine (ACSM) [33] established recommendable health standards of PA practice, measured in parameters of frequency, duration, intensity, type and total quantity of PA, beyond the physical activities of daily living, with the aim of improving health and decreasing morbidity and premature mortality, especially in men, who show worse health behavior and lower life expectancy than women [50,51,52,53]. Table 1 shows an adaptation of these recommendations. 

However, the WHO identified sedentary behaviors and low levels of PA during the quarantine that may have negative effects on the health of the population. Therefore, it gathered guidelines of PA and relaxation techniques for healthy people to stay active during the confinement, helping to maintain calmness and protect health [54]. For their part, the ACSM recommended maintaining moderate practice during the quarantine period, as it helps to strengthen the immune system against SARS-CoV-2 [42], and performing aerobic exercises and resistance training indoors: squats, jumps and lunges, push-ups, walking briskly around the house, going up and down the stairs, dancing, rope jumping and practicing yoga. They also recommend, whenever possible, walking or running outdoors, cycling, gardening and playing family games, always including exercises that involve muscles of the lower and upper body and limbs, using elastic bands or even backpacks, books and water bottles. They advise using technological tools and the counseling of a professional to improve the performance of these activities in the home environment [55]. These recommendations are listed in Table 2. Other authors, such as [56], recommend practices adapted to the child and adolescent population.

Furthermore, it is important to consider the effects of the confinement on PA practice and, consequently, on health as a function of gender (men/women), in order to determine the existence of differences in this regard. The experience of past outbreaks demonstrates the importance of incorporating a gender analysis to identify differences and prepare effective responses in health intervention, promoting gender and health equity [57,58]. Therefore, this is an opportunity to analyze the current situation from the gender perspective, since, in general, young, adult and elder women are less active than young, adult and elder men [40,59,60,61,62,63]. This difference may have increased during the confinement and, thus, the immune response to the virus, body weight, cardiopulmonary conditioning, blood glucose and insulin levels, lipid metabolism, etc. associated with a lack of or scarce PA may have also changed differently between the two genders. 

In the EU, men practice more PA and do so more frequently than women, although the gender divide decreases with age, especially from the age of 55 years. Moreover, there are more women than men who never practice any sport, with the corresponding repercussions on health [64,65]. Regarding the intensity of PA practice, it is also men who are more inclined to perform the vigorous PA that is recommended by the health authorities (18% of men vs. 11% of women), whereas 51% of men vs. 65% of women do not practice any vigorous PA, which is a striking difference [64,65]. Despite this, European men show less healthy behaviors (alcohol consumption, smoking, overweight, worse diet) and worse health results [52] than women and men in Spain [50,51].

The PA patterns are also different between men and women. Regarding the PA format, men usually practice PA in sports clubs, gyms, at home and outdoors, whereas women mainly exercise at home and outdoors [29,32]. Similarly, the motivation for PA practice is different between men and women. The former are motivated by having fun, spending time with friends and competing, while women prefer to practice PA for the sake of health, relaxation and beauty [64]. Differences in formats and motivations of practice between genders may have influenced the quantity of PA performed by both genders during the pandemic and, consequently, their health. Men and women coincide in the main barrier that limits PA practice, i.e., the lack of time (between 55% and 51% state this reason); however, there is a slightly more important barrier for women, which is the lack of interest or motivation (22% vs. 19%) [64].

Specifically in Spain, the survey on sports habits among Spanish people and the Spanish national survey on health showed similar data. There are considerable differences between men and women, and, although the divide between them has decreased since 2010, the percentage of men and women who practice PA is 59.80% and 47.50%, respectively [66]. Moreover, according to the Spanish national health survey, more women ignore the recommendations of the WHO regarding PA (37% women vs. 34% men), and women perform less of the recommended PA vigorous intensity (18.40% women vs. 30.30% men) [67]. With respect to the type of practice, men prefer group and outdoor sports, or bodybuilding, whereas women opt for any type of aerobic exercise, with or without music, swimming and indoor sports [66].

This study also gathers the motivations and barriers to practicing PA. Being fit is the main motivation to practice PA for both genders, especially for women (32.70%). The second motivation for men is to have fun (27%), whereas this is important for only 18.40% of women. In addition, women also practice PA to stay healthy and relax. Regarding the barriers to PA practice, the lack of time and the lack of interest are in the leading positions. 

Therefore, those differences in quantity, intensity, type and means of practicing PA between genders may have increased during the confinement, with greater negative consequences for women’s health. 

Thus, the general aim of this study was to analyze the impact of the COVID-19 confinement on PA habits as a function of gender (men/women) in Spain, considering PA practice as a health factor. Since there have been no studies on this topic to date due to the recentness of the situation, we considered it relevant to describe such impact before carrying out more thorough analyses. This study specifies the following objectives and hypotheses:

Objective 1. To compare PA practice before and during the confinement, based on gender (men/women) and the standards recommended by the WHO and the ACSM. To attain this objective, the following working hypotheses were proposed:

**Hypotheses** **1a** **(H1a).** *During the confinement, women maintained the quantity of PA practice that they performed before the confinement.*

**Hypotheses** **1b** **(H1b).** *During the confinement, men maintained the quantity of PA practice that they performed before the confinement.*

**Hypotheses** **1c** **(H1c).** *During the confinement, women maintained the intensity of PA practice that they performed before the confinement.*

**Hypotheses** **1d** **(H1d).** *During the confinement, men maintained the intensity of PA practice that they performed before the confinement.*

**Hypotheses** **1e** **(H1e).** *During the confinement, women continued to perform the same type of PA practice that they did before the confinement.*

**Hypotheses** **1f** **(H1f).** *During the confinement, men continued to perform the same type of PA practice that they did before the confinement.*

Objective 2. To compare whether the PA standards recommended by the WHO and the ACSM were met during the confinement, based on gender (men/women).

**Hypotheses** **2** **(H2).** *During the confinement, the PA standards recommended by the WHO and the ACSM were met based on gender (men/women).*

Objective 3. To compare the difference in PA practice between men and women during the confinement.

**Hypotheses** **3a** **(H3a).** *The variation in the quantity of PA practiced during the confinement was the same for both women and men.*

**Hypotheses** **3b** **(H3b).** *The variation in the intensity of PA practiced during the confinement was the same for both women and men.*

**Hypotheses** **3c** **(H3c).** *The variation in the type of PA practiced during the confinement was the same for both women and men.*

Objective 4. To determine, based on gender, the effect that the confinement had on PA practice in those who did not practice PA before the confinement.

**Hypotheses** **4a** **(H4a).** *Women who did not practice PA before did so during the confinement.*

**Hypotheses** **4b** **(H4b).** *Men who did not practice PA before did so during the confinement.*

Objective 5. To determine, based on gender, the format of PA practiced during the confinement.

**Hypotheses** **5a** **(H5a).** *Women performed the same autonomous PA as men.*

**Hypotheses** **5b** **(H5b).** *Women performed the same PA as men through virtual classes.*

## 2. Materials and Methods 

### 2.1. Participants

The study was focused on the general Spanish population in 2019, composed of 47,431,256 inhabitants—49% males and 51% females [68]. Although the sampling was not representative, the obtained and analyzed sample fairly approached these population figures, consisting of 1046 participants of both genders (48.57% men and 51.43% women), with an average age of 40 years (SD = ±13.35). Regarding their education level, 81.07% had higher education, 16.63% had secondary education, 2.10% had primary education and only two participants had no education. Responses to the survey came from almost every autonomous community.

### 2.2. Instrument

The study was conducted using a descriptive quantitative methodology based on random, non-purposive sampling. 

Once the objectives were set, an ad hoc questionnaire was created, which included, in addition to the sociodemographic data, aspects related to PA habits before and during the confinement caused by the COVID-19 pandemic (Appendix A). For the data collection, the questionnaire was divided into different areas, taking different scales as a reference: multiple-choice and categorization through a 5-item Likert scale, considering that the items with odd scoring are the most popular [69].

The questionnaire was validated by an expert panel, selected in accordance with the requirements proposed by [70]. Specifically, the panel consisted of 12 experts in the scope of teaching and health, with equal participation in terms of gender. Of these 12 experts, 4 were active professors with over 10 years of experience (in the field of science of physical activity and sport, psychology and new technologies), 2 were experts from companies of the field of new technologies, 2 were managers of municipal services and the remaining 2 experts were owners of fitness centers and gymnasiums. In addition to these 12 experts, another 2 people, who were non-experts in the area of PSA, also participated in the expert panel; one of them practiced PA regularly, whereas the other person did not. 

### 2.3. Procedure

The information-gathering instrument was a form applied through the Google Forms platform, ensuring confidentiality and anonymity at all times. This instrument allowed us to collect data regarding the actions, opinions and thoughts of the surveyed participants [71]. Moreover, the administration of the online questionnaire was not only the best option but also the only one to obtain the information, due to the confinement.

Informed consent was obtained from the participants prior to the data collection, which was carried out by having participants answer a question at the beginning of the questionnaire. The participants were informed that all information gathered was to be used solely for scientific purposes.

Ethical approval was requested from the Ethics and Human Research Committee of the University of Valladolid (Spain). The principles of the Declaration of Helsinki [72] were followed for this type of research.

The data were collected from 10 April to 10 May 2020, a period when PA could not be practiced outside of the home environment. The link to the referred questionnaire was shared via different electronic applications, social networks and email with diverse associations, institutions and companies of both sport and non-sport sectors, with the aim of reaching their members. Some of these entities were sport federations (national and regional), business and civil associations, universities, public and private schools, friends, relatives, etc.

Of the data collected, 39 questionnaires were discarded due to incorrect completion (21 females, 18 males).

### 2.4. Data Analysis

Once the database was filtered, the data were treated using the statistical package IBM SPSS Statistics (v.26). The obtained answers were subjected to Kolmogorov–Smirnov tests, which showed that the study variable did not meet the normality assumption. Therefore, since it was not possible to use parametric techniques to verify the hypothesis (Student’s *t*-test), the non-parametric alternative was selected, i.e., the Wilcoxon’s rank test for paired samples. In all cases, a significance level of *p* ≤ 0.05 was established. Moreover, descriptive measures were obtained, i.e., mean, standard deviation or frequency, depending on the nature of the data. 

## 3. Results

In this section, we address the objectives and research hypotheses that were previously set.

With respect to objective 1, PA practice before and during the confinement was analyzed for the two groups (men and women) regarding the PA standards recommended by the WHO and the ACSM. Specifically, we analyzed the parameters of time (quantity), intensity and type of PA recognized by these organizations. As for frequency (daily/weekly PA habits), due to the temporal conditions of the confinement, no question in this regard was included in the questionnaire, since it was a very short analytical period and, initially, the participants would not have been able to provide a valid answer. 

Table 3 shows the results obtained for the first two parameters, separated by gender (women *n* = 538 and men *n* = 508), both before and during the confinement. It is worth mentioning that the information for PA quantity was gathered according to categories “1. None (no PA done)”, “2. Few (under 3 h per week)”, “3. Some (3 h to 5 h 59 m per week)”, “4. Enough (6 h to 8 h 59 m per week)” and “5. A lot (more than 9 h per week)”, whereas PA intensity was measured as “1. None”, “2. Light”, “3. Moderate” and “4. Vigorous”.

Table 3 shows that both men and women decreased their PA quantity and intensity during the confinement. In fact, in terms of quantity, both groups moved from category “3. Some (3 h to 5 h 59 m)” to category “2. Few (under 3 h)”. PA intensity was also lower in both groups, which also posed a shift to an inferior category.

Furthermore, for the significance level established, the variation (decrease) was significant in both quantity and intensity, regardless of gender. The PA quantity practiced by the women (H1a) during the COVID-19 confinement (M = 2.62) was lower than before the confinement (M = 3.02). The PA intensity (H1a) during the confinement (M = 2.62) was also lower than before the confinement (M = 3.08). Similarly, the PA practiced by the men during the confinement (H1b) was lower in quantity (M = 2.79) with respect to the PA practiced before the confinement (M = 3.50), and the PA intensity (H1d) during the confinement (M = 2.90) was also lower than before the confinement (M = 3.45).

To analyze the type of PA, the questionnaire asked about eight sports disciplines (Table 4 caption), whose valuations were grouped into the terms “None”, “Few–Some” and “Enough–A lot”, before and during the confinement. For a better analysis, Table 4 groups these disciplines into the four types recommended by the ACSM [33].

**Figure 1:**
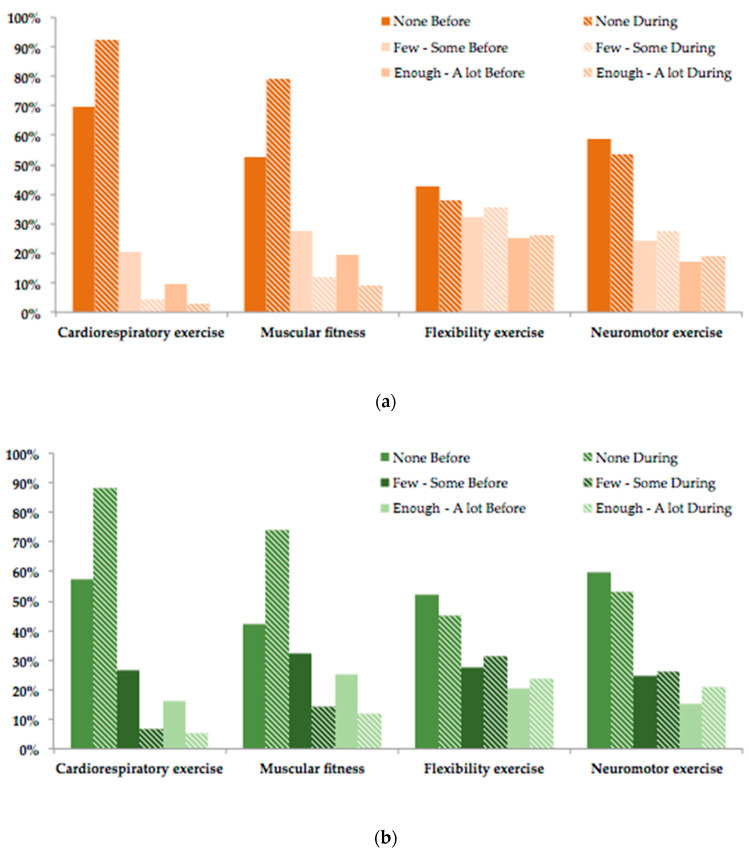
Comparison of the type of PA practiced before and during the confinement, based on [33], in women (**a**) and men (**b**).

The previous data are graphically represented in Figure 1, which shows a similar change in the type (H1e and H1f) of PA practice in men and women: there was a significant increase during the confinement in those who practiced no (“None”) cardiorespiratory exercise (up to approximately 90%) and muscular fitness exercise (around 75%); however, there was a decrease for both men and women in those who practiced no (“None”) flexibility and neuromotor exercises, indicating that they practiced more PA during the confinement than before it.

With respect to objective 2 (H2), the previous results allow for verifying whether the PA standards recommended by the WHO and the ACSM were met during the confinement, based on gender (men/women). Regarding PA quantity, it was recommended to practice PA for between 200 and 400 min per week during the confinement [73,74]. The results show that both genders carried out under 180 min of PA, especially women. As for intensity, the recommended health standard was to maintain moderate practice during the quarantine. Both genders decreased the PA intensity to light practice, although the men were close to the recommended moderate intensity. With respect to the ideal type of PA practice, it was recommended to continue carrying out exercises of the four types indicated; however, mainly cardiovascular exercise and muscular fitness PA decreased in both genders.

Previously, a decrease was observed in quantity and intensity, for both men and women (Table 3). Objective 3 states the need to verify the significance of such variation (Table 5) in PA standards according to gender.

For the significance level established, the differences obtained for the confinement period in both quantity and intensity are significant (Table 3), although these differences were also significant before the confinement; thus, it can be concluded that such differences remain the same. It can be observed that men practiced more PA than women on average, both before and during the confinement, and that the intensity was also greater before and during the confinement in men. However, despite the fact that the two parameters decrease during the confinement in the two groups (Table 5), the observed decrease is always lower in the case of women, both in quantity (H3a) (average decrease in women −0.40; average decrease in men −0.71) and intensity (H3b) (average decrease in women −0.46; average decrease in men −0.55), being especially significant in the case of quantity (H3a) (*p* < 0.000).

**Figure 2:**
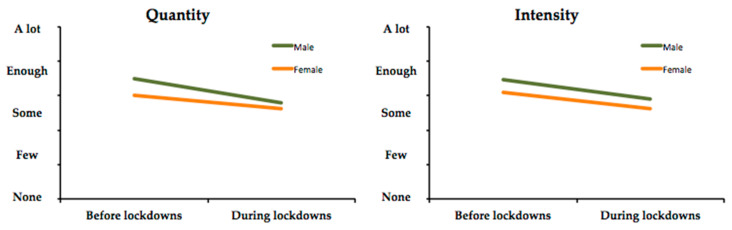
Comparative variation in quantity and intensity based on gender.

This variation, which is similar in terms of intensity and especially different regarding quantity based on gender, is clearly represented in Figure 2.

With respect to the type of PA (H3c), as was previously commented, the change in this parameter was similar for both genders: the practice of cardiorespiratory exercise and muscular fitness decreased, and the practice of flexibility exercise and neuromotor or functional training increased. Specifically, the differences between during and before the confinement for each type of practice based on gender are shown in Table 6.

As can be observed, there was an increase of 22.54% and 30.46% for women and men, respectively, who did not practice cardiorespiratory exercise, with an increase of 26.17% and 31.50% for women and men, respectively, who did not practice muscular fitness. Moreover, with respect to these two types of PA practice, there was a decrease in those who performed this type of exercise with variable intensity (in the case of “Few–Some”, the decrease was similar in men and women, whereas in the case of “Enough–A lot”, the decreases were more significant in men).

Objective 4 was to determine, as a function of gender, the effect of the confinement on PA practice in people who did not practice any PA before. The results for women (H4a) and men (H4b) are shown in Table 7, which only considers the surveyed people who did not practice PA before the confinement. In this respect, it is important to mention that the sample is especially small in this case (women *n* = 38, men *n* = 16); nevertheless, it was decided to carry out the analysis with the available data. 

The results show, for the significance level established, that there was a significant increase in both men and women who started practicing PA during the confinement. From practicing no PA (“None”), they shifted to practicing a “Few” hours of PA (M = 1.80 in the case of women and M = 1.50 in the case of men). Regardless of gender, all the surveyed individuals fell into the category of 0–3 h of PA practice per week. It is worth mentioning that women who adopted an active lifestyle did so in a slightly higher quantity than men, since their average was in the higher part of this category.

Lastly, objective 5 was to explore, as a function of gender, the format of PA practiced during the confinement, by analyzing the answers in this regard. Specifically, two possible answers were analyzed: “1. On my own (at home or outdoors/in the garden)” (H5a) and “2. Through virtual classes (individual, personal trainer or sports organization)” (H5b) (Table 8).

The obtained results were statistically significant in both cases. Regarding the first option (PA on their own at home or outdoors/in the garden), it is observed that, during the confinement period, both men and women used this format for “Some” hours per week. Particularly, men (M = 3.58) practiced more PA on their own (at home or outdoors/in the garden) than women (M = 3.18). However, in the case of PA through virtual classes, during the confinement, women used this format (M = 2.81) much more frequently than men (M = 1.94). On average, men used this format for a “Few” hours per week, whereas women fell into the category of “Enough”.

## 4. Discussion

The spread of COVID-19 throughout the world has forced governments to make very restrictive decisions. In the case of Spain, on 14 March 2020, a strict confinement of the entire population was enforced, which lasted one month and a half. This movement limitation led to a radical modification in the habits of PA practice, resulting, in many cases, in a significant decrease in the quantity, intensity and type of PA practiced [14,15], as well as a change in the format in which PA was practiced. 

Consequently, the standards of PA practice recommended by the different organizations [33,36] through their public health programs were not met and, thus, the positive benefits to physical, social and emotional health were seriously compromised. Moreover, it is worth highlighting that the confinement had different effects on women and men [57] and, therefore, on their practice levels. This fact justifies the realization of investigations that consider possible gender differences in order to develop effective intervention responses, which was the focus of the present study.

In general, both men and women showed a significant decrease in both the quantity and intensity of PA during the confinement (H1a, H1b, H1c, H1d), although men tended to be more active than women, which is in line with the results reported in [40,59,60,61,62,63]. In fact, regarding PA quantity, both groups shifted from practicing “Some” hours of PA per week (between 3 h and 5 h 59 m) to a “Few” hours of PA per week (less than 3 h). The intensity was also lower in both cases, with a change to an inferior category, i.e., from “moderate” to “light”. However, the health authorities recommended even increasing the quantity of PA and maintaining moderate intensity during the confinement [54,55].

The observed changes in the type of PA practiced [33] are similar in both genders (H1e, H1f), with a decrease in cardiorespiratory and muscular activity. Likewise, the results allow us to assert that, during the confinement, the recommended PA standards (H2) were not met, although flexibility and neuromotor exercises increased. Therefore, it can be affirmed that hypothesis 1 is not fulfilled, neither for men nor for women.

These results are in line with those of other studies conducted in university populations [27], older adults [75] and in the general population [76]. Although the media repeatedly state that PA practice had become one of the routine activities during the confinement, especially in women [77], and other investigations [32] agree with such statements, the data analyzed in the present study do not confirm this reality. 

Moreover, our results allow us to assert that, during the confinement, the PA standards recommended by [33,36] were not met (H2), considering that, given the living conditions in this period, the quantity of PA needed to be increased and the intensity and type of PA needed to be maintained [43,54,73,74], which is in line with [75]. This failure occurred especially in the intensity of PA practiced by women. Therefore, hypothesis 2 is not fulfilled.

As was expected, the variations in PA when comparing the data from before and during the confinement were negative, both for women and men, although the decreases in quantity (H3a), intensity (H3b) and type (H3c) were more pronounced in the case of men and statistically greater regarding quantity (H3a). This indicates that men reduced their hours of PA to a greater extent than women and demonstrates the existence of different motivations for the practice of PA according to gender, being more social, ludic and competitive for the former and more individualistic and introspective (inward focus) for the latter. Furthermore, the fact that women prefer non-competitive and relaxing activities [63,66,67] led to a smaller decrease in PA quantity with respect to men. Thus, it can be asserted that hypothesis 3 is not fulfilled. 

These findings show a clear contradiction with other studies, such as the one conducted by [74], who reported that the group of people who practiced little or no PA decreased throughout the second and third weeks of the confinement. 

Interestingly, men and women who did not practice any PA before the confinement decided to start practicing some PA during the confinement (H4a, H4b). Specifically, more women who were passive before the confinement started practicing PA during the confinement (more than twice as much as men), which could indicate greater adaptability of women to new circumstances. Therefore, it can be asserted that hypothesis 4 is fulfilled, which is in line with the results of similar studies conducted in Italy and Canada [14,15]. 

Regarding the format of PA practice during the confinement, the results show that men practiced more PA on their own than women (H5a), who prefer directed activities [78], indicating that they are more autonomous when practicing PA (hypothesis H5a not fulfilled), although women use virtual classes to a greater extent than men (hypothesis H5b not fulfilled). Thus, it could be asserted that women made better use of the digital services offered by many organizations to respond to the situation [16,22,23,24] compared to men, which supports the results of hypothesis 4 regarding the greater capacity of women to adapt to these circumstances. This could explain the lower decrease in the quantity of PA practice of women compared to men during the confinement, since men preferentially practice team sports, in the gym or outdoors, and they like to compete [29,32,79,80], which was not possible during the confinement. 

The scientific evidence is clear: the regular practice of sports and exercise contributes to wellbeing and quality of life [81], being a key instrument to promote health, prevent health risks, reduce socio-sanitary costs, activate the social and economic scopes of life, foster sustainable development and preserve the planet [82,83,84]. It could be asserted that this key point (PA and sports practice), in view of the obtained results, was seriously threatened by the COVID-19 confinement situation to which the Spanish population was subjected, especially in women, considering that, already before the pandemic, they claimed to be less active than men [40,56,57,59,60,61], which would result in even more harmful effects on health, although they show better general health behaviors (lower consumption of toxic substances, better diet, less overweight) and greater life expectancy [50,51]. 

### Limitations and Future Research Lines 

This study has some limitations that must be considered in future investigations, such as the small sample size. However, given the recentness of the topic, this transversal and non-experimental study describes, reflects and guides the authorities about what can happen in the population in the case of further confinement scenarios and, therefore, provides valuable information about an exceptional moment in the history of the 21st century: the beginning of the COVID-19 pandemic. Therefore, this study provides data to administrations for the promotion of positive policies oriented toward reducing the levels of physical inactivity, and it would be interesting to monitor the evolution of PA practice in the post-COVID-19 period. It is necessary to study the impact of the use of new technologies and, thus, analyze the divide that the lack of these may cause in terms of physical inactivity, which can become a limiting agent to maintaining the standards of wellbeing. Other relevant aspects worth studying are the combination of the variables of PA practice in relation to the influence of mood and emotional and/or emotivational aspects on sports practice. In addition, further research related to diet and eating habits in stressful situations must also be carried out, since these can lead to weight gain, thus seriously damaging the health of individuals. Consequently, future studies should carry out interventions based on gender to determine whether the intentions of PA practice increase in men and women according to the variation in the practiced PA type, quantity, intensity and format.

## 5. Conclusions

The scientific community has warned about the different direct and indirect effects of COVID-19 [57] as a function of gender. One such effect is the impact of the COVID-19 confinement on PA practice as a health agent, in men and women, which was described and evidenced in the present study. The results show that the confinement induced a significant decrease in the quantity and intensity of PA practice in both genders, as well as a decrease in some types of recommended practices, which had a negative effect on health. However, it is necessary to highlight that COVID-19 had a greater negative impact on women. 

The health alarm situation has modified the participation of both genders in PA practice, particularly jeopardizing the advances of female PA practice in the last decade [85]. It is necessary to delve into the impact of such a situation on both genders, not only in terms of health but also in terms of the permanence of sport consumption habits and the capacity of men and women to adapt to the new normality in sports. Such knowledge will allow us to encourage institutions to implement policies and practices that consider gender, the different responses that men and women have shown throughout the confinement regarding their PA practices and the consequences of such responses, in order to diversify the offer of PA and reduce inactivity. To sum up, PA must be made accessible to both genders, with innovative strategies to face the new normality in the practice of PA, since it is a healthy agent in more or less restrictive confinement situations and beyond. 

## Figures and Tables

**Table 1:** Adapted from Physical Activity Guidelines [33,36].

Type	Intensity	Frequency	Duration	Quantity	Examples
Cardiorespiratory exercise	Moderate	≥5 d·wk	≥30 min·d Sessions of at least 10 min	≥150 min·wk Additional health benefits are obtained with 300 min ∙ wk	Rhythm, aerobic exercises involving large muscle groups
	Vigorous	≥3 d·wk	≥20 min·d	≥75 min·wk To be more effective increase to 150 min· wk	
	Or a combination of moderate and vigorous exercise on ≥3–5 d·wk				
Muscular fitness (muscular strength, endurance and power)	Should be tailored to the individual’s experience	2–3 d·wks with at least 48 h separating the exercise training sessions for the same muscle group	A reasonable rest interval	2–4 sets 8–12 repetitions per set 8–10 exercises for session	Training involving each major muscle group. Multi-joint exercises (stair climbing, carrying bags of groceries).
Flexibility exercise	Stretch to the point of tightness or slight discomfort	≥2–3 times·wk Most effective when performed daily	30–60 s/ex Series 10–30 s Adjusting time/duration and repetitions according to individual needs	2–4 series	Exercises targeting the major muscle tendon units should be performed (postural stability and flexibility exercises)
Neuromotor exercise	Not determined	≥2–3 d·wk	≥20–30 min· d	≥60 min·wk	Involving balance, agility, coordination, gait

Note: d: days; wk: week; min: minute; ex: exercise.

**Table 2:** Guidelines during quarantine according to [54,55].

Recommendations of Physical Activity during Quarantine according to WHO and ACSM
150–300 min per week of moderate-intensity aerobic physical activity and 2 sessions per week of muscle strength training
Walk briskly around the house. Dance. Jump rope. Walk up and down the stairs
7-Minute Workout app
Yoga
Simple muscle strengthening exercises around your house: squats or sit-to-stands from a sturdy chair, push-ups against a wall, the kitchen counter or the floor, lunges or single leg step-ups on stairs
Do not sit all day
Relaxation techniques

**Table 3:** Quantity and Intensity of physical activity (PA) before and during the confinement.

		M	SD	Wilcoxon W	Z	Sig.
Female (*n* = 538)						
Quantity	Before	3.02	1.16	17,850.000	−7.458	0.000
	During	2.62	1.01			
Intensity	Before	3.08	0.81	8629.500	−10.729	0.000
	During	2.62	0.81			
Male (*n* = 508)						
Quantity	Before	3.50	1.30	9049.000	−11.283	0.000
	During	2.79	1.17			
Intensity	Before	3.45	0.77	4676.500	−11.655	0.000
	During	2.90	0.92			

**Table 4:** Comparison of the types of PA practiced before and during the confinement.

	BEFORE		DURING	
	Female	Male	Female	Male
**Cardiorespiratory exercise (1)**				
None	69.89%	57.58%	92.43%	88.04%
Few–Some (<6 h)	20.45%	26.38%	4.55%	6.84%
Enough-A lot (>6 h)	9.67%	16.04%	3.02%	5.12%
**Muscular fitness (2)**				
None	52.86%	42.44%	79.03%	73.94%
Few-Some (<6 h)	27.58%	32.40%	11.93%	14.06%
Enough–A lot (>6 h)	19.55%	25.16%	9.03%	12.01%
**Flexibility exercise (3)**				
None	42.75%	51.97%	38.10%	44.98%
Few-Some (<6 h)	32.25%	27.56%	35.69%	31.20%
Enough–A lot (>6 h)	25.00%	20.47%	26.21%	23.82%
**Neuromotor exercise (4)**				
None	58.74%	59.78%	53.47%	52.95%
Few–Some (<6 h)	24.29%	24.80%	27.51%	26.25%
Enough–A lot (>6 h)	16.98%	15.42%	19.02%	20.80%

Note: (1) cycling, swimming, racket sports, team sports; (2) cycling, outdoor sports, racket sports, team sports, gym sports; (3) gym sports, light gymnastics; (4) gym sports and light gymnastics.

**Table 5:** Comparison of the variation between men and women, before and during the confinement, in quantity and intensity.

Female = 538 Male = 508	Group	M	SD	Mann–Whitney U	Wilcoxon W	Z	Sig.
Quantity_ Variation	Female	−0.40	1.21	154.937	299.928	3.883	0.000
	Male	−0.71	1.25				
Intensity_ Variation	Female	−0.46	0.87	141.996	286.987	1.174	0.241
	Male	−0.55	0.91				

**Table 6:** Differences (during-before) by gender in each type of PA practice.

	None		Few–Some		Enough–A lot	
	Female	Male	Female	Male	Female	Male
DURING-BEFORE Cardiorespiratory exercise	22.54%	30.46%	−15.89%	−19.54%	−6.64%	−10.93%
Muscular fitness	26.17%	31.50%	−15.65%	−18.35%	−10.52%	−13.15%
Flexibility exercise	−4.65%	−6.99%	3.44%	3.64%	1.21%	3.35%
Neuromotor exercise	−5.27%	−6.82%	3.22%	1.44%	2.04%	5.38%

**Table 7:** Quantity of PA, individuals who did not practice PA before the confinement.

		M	SD	Wilcoxon W	Z	Sig.
Female (*n* = 38)	Before	1.00	0.00	21.000	4.042	0.000
	During	1.80	0.85			
Male (*n* = 16)	Before	1.00	0.00	28.000	2.530	0.011
	During	1.50	0.63			

**Table 8:** PA practice through the autonomous format and virtual classes during the confinement.

N = 1046 Female = 538 Male = 508	Group	M	SD	Mann–Whitney U Test			
				Mann–Whitney	Wilcoxon W	Z	Sig.
(1)	Female	3.18	1.41	119.721	264.712	−3567	0.000
	Male	3.58	1.42				
(2)	Female	2.81	1.56	179.875	324.866	9385	0.000
	Male	1.94	1.31				

Note: (1) “On my own (at home or outdoors/in the garden)” and (2) “Through virtual classes (individual, personal trainer or sports organization)”.

## Appendix A

Sociodemographic data (English version)

1. Gender: Man/Woman
2. Age (years)
3. Province (selection of the corresponding Spanish province)
4. Education level (no education/Primary Ed./Secondary Ed./Higher Ed.)

Aspects of physical activity

5. Physical activity intensity (1. None/2. Smooth/3. Moderate/4. Intense)

Before COVID-19

During COVID-19

6. Weekly hours of physical activity (1. None (cero)/2. Few (<3 h)/3. Some (between 3h and 5h59′)/4. Enough (between 6h and 8h59′)/5. A lot (over 9h))

Before COVID-19

During COVID-19

7. To what extent (1. None/2. Few/3. Some/4. Enough/5. A lot) did you practice the following activities? (cycling, swimming, outdoor sports, racket sports, gym sports, smooth gymnastics, e-sports)

Before COVID-19

During COVID-19
